# Double trouble: the impact of multimorbidity and deprivation on preference-weighted health related quality of life a cross sectional analysis of the Scottish Health Survey

**DOI:** 10.1186/1475-9276-12-67

**Published:** 2013-08-20

**Authors:** Kenny D Lawson, Stewart W Mercer, Sally Wyke, Eleanor Grieve, Bruce Guthrie, Graham CM Watt, Elisabeth AE Fenwick

**Affiliations:** 1 Health Economics and HTA, Institute of Health & Wellbeing, University of Glasgow, 1 Lilybank Gardens, Glasgow G12 8RZ, UK; 2 General Practice & Primary Care, Institute of Health & Wellbeing, University of Glasgow, 1 Horselethill Road, Glasgow G12 9LX, UK; 3 Institute of Health & Wellbeing, University of Glasgow, 25-28 Bute Gardens, Glasgow G12 8RS, UK; 4 Quality Safety and Informatics Research Group, Population Health Sciences Division, Medical Research Institute, University of Dundee, The Mackenzie Building, Kirsty Semple Way, Dundee DD2 4BF, UK

**Keywords:** Multimorbidity, Preference_Weighted Health Related Quality of Life (PW_HRQoL), Deprivation, Inequality

## Abstract

**Objective:**

To investigate the association between multimorbidity and Preference_Weighted Health Related Quality of Life (PW_HRQoL), a score that combines physical and mental functioning, and how this varies by socioeconomic deprivation and age.

**Design:**

The Scottish Health Survey (SHeS) is a cross-sectional representative survey of the general population which included the SF-12, a survey of HRQoL, for individuals 20 years and over.

**Methods:**

For 7,054 participants we generated PW_HRQoL scores by running SF-12 responses through the SF-6D algorithm. The resulting scores ranged from 0.29 (worst health) to 1 (perfect health). Using ordinary least squares, we first investigated associations between scores and increasing counts of longstanding conditions, and then repeated for multimorbidity (2+ conditions). Estimates were made for the general population and quintiles of socioeconomic deprivation. For multimorbidity, the analyses were repeated stratifying the population by age group (20–44, 45–64, 65+).

**Results:**

45% of participants reported a longstanding condition and 18% reported multimorbidity. The presence of 1, 2, or 3+ longstanding conditions were associated with average reductions in PW_HRQoL scores of 0.081, 0.151 and 0.212 respectively. Reduction in scores associated with multimorbidity was 33% greater in the most deprived quintile compared to the least deprived quintile, with the biggest difference (80%) in the 20–44 age groups. There were no significant gender differences.

**Conclusions:**

PW_HRQoL decreases markedly with multimorbidity, and is exacerbated by higher deprivation and younger age. There is a need to prioritise interventions to improve the HRQoL for (especially younger) adults with multimorbidity in deprived areas.

**Box 1:**

What Is Known?

Prevalence and premature onset of multimorbidity increases as socioeconomic position worsens. Previous studies have investigated the effect of multimorbidity on Health Related Quality of Life (HRQoL) on separate physical and mental health states. There is limited data on how HRQoL falls as the number of conditions increase, and how estimates vary across the general population.

Leaving physical and mental health as separate categories can inhibit assessment of overall HRQoL. The use of a Preference_Weighted Health Related Quality of Life (PW_HRQoL) score provides a single summary measure of overall health, by weighting mental and physical states by their perceived importance as part of overall HRQoL. The use of a single score enables a simple and consistent assessment of the impact of conditions and how this varies across the population. Economists term PW_HRQoL scores health utilities.

What this study adds?

This is the first study to estimate how the impact of multimorbidity on PW_HRQoL scores varies by age group and socioeconomic deprivation. Multimorbidity has a substantial negative impact on HRQoL which is most severe in areas of deprivation, especially in younger adults.

Measuring the burden of multimorbidity using PW_HRQoL provides consistency with how economists measure HRQoL; changes in which can be used in economic evaluation to assess the cost effectiveness of interventions.

## Background

The prevalence of longstanding conditions and multimorbidity is increasing globally, primarily as a result of increased life expectancies [1]. It is of major concern to policy makers and wider society because of the associated, rising, cost and complexity of health care and the changes to the organisation and delivery of care that will be necessary to manage it effectively. A recent Cochrane review, of interventions in patients with multimorbidity in primary care and community settings, reported a lack of evidence-based interventions to help people to better manage their conditions [2].

The association between multimorbidity on Health Related Quality of Life (HRQoL) remains under researched [3-5]. A previous review and subsequent study found a negative association between multimorbidity and HRQoL [6,7]. However, it is largely unknown how the impact varies within the general population, with no studies assessing the impact of multimorbidity by socioeconomic deprivation [8]. There is also limited understanding regarding the extent to which overall HRQoL falls as the number of conditions increase, and as case-mix changes.

A key issue is how HRQoL is measured. Commonly used instruments leave mental and physical health as separate dimensions. This can be important for exploring which aspects of health may suffer as a result of experiencing longstanding conditions; however, it inhibits direct comparisons of the overall severity of different conditions, and the extent to which overall HRQoL falls as the number of conditions increase. A single score summarising overall HRQoL is required. Preference Weighted Health Related Quality of Life (PW_HRQoL) combines mental and physical health into a single summary score, which can range from 0 (death) to 1 (perfect health), and certain scores also measure states worse than death. Instruments that can be used to create PW_HRQoL scores include the SF-6D [9], EuroQol-5D [10], and Health Utilities Index [11]. PW_HRQoL scores are referred to as health utilities by economists. Measuring the burden of multimorbidity using PW_HRQoL provides consistency with how economists measure HRQoL; changes in which can be used in economic evaluation to assess the cost effectiveness of interventions.

We are only aware of two studies to date that have investigated the association between PW_HRQoL scores and increasing numbers of longstanding conditions: one in a US general population [12] and the other in two General Practices in Manchester, England [13]. Consistent findings were that PW_HRQoL scores fell markedly as the number of conditions increased. However, both studies were limited in that they considered only cardiovascular and respiratory conditions. To strengthen generalisations regarding the associations of multimorbidity and HRQoL it is important to include a wider set of conditions and undertake measurement in the general population.

The studies also did not consider the associations between multimorbidity, HRQoL and socioeconomic deprivation. It is known that multimorbidity is more common and develops earlier in deprived areas, and that people living in deprived areas experience a range of problems that add to the considerable complexity of clinical and self-management support of multimorbidity [14]. A previous study found that deprived patients may suffer from reduced levels of personal capacity or community support with which to manage or mitigate the impact of multimorbidity [14]. Further, the existence of the ‘inverse care law’ [15], where the availability of services does not reflect the greater needs of deprived populations may result in insufficient health care resources to help people manage their conditions.

The main objective of this study was to use data from the 2003 Scottish Health Survey to examine whether and to what degree PW_HRQoL in those living with multimorbidity varies by socioeconomic deprivation and age. It was hypothesised that the impact of multimorbidity on PW_HRQoL would increase as socioeconomic deprivation worsens.

## Methods

### Source data

The Scottish Health Survey (SHeS) is a periodic cross-sectional survey of the health and health behaviours of the Scottish population. The survey uses multi-stage stratified probability sampling of eligible households with the objective to obtain a representative sample of the general population across Scotland [16]. As part of the survey, individuals were asked to self-report whether they suffer from longstanding illnesses. The question asked was: ‘*Do you have any long*-*standing illness*, *disability or infirmity*? *By long*-*standing I mean anything that has troubled you over a period of time*, *or that is likely to affect you over a period of time*.’ We use the term longstanding conditions, instead of longstanding illness hereon. The survey can record up to 40 longstanding conditions, commensurate with ICD-10 classifications, of which individuals can report up to six [17]. The SHeS then groups these 40 conditions into 15 systems of the body (Table 1), also commensurate with ICD-10 coding [17].

**Table 1:** Longstanding conditions included in Scottish Health Survey 2003

**Type of longstanding condition**	**Grouped (system of body)**	**Number/% within grouped conditions**
1 Cancer (neoplasm) including lumps, masses, tumours & growths	Neoplasms & begin growths	
2 Diabetes. Incl. Hyperglycemia	Endocrine and metabolic	443 (6.3%)
3 Other endocrine/metabolic		
4 Mental llness.anxiety/despression/nerves		275 (3.9%)
5 Mental handicap		
6 Epilepsy/fits/convulsions	Nervous system	289 (3.9%)
7 Migraine/headaches		
8 other problems of nervous system		
9 Cataract/poor eye sight/blindness	Eye complaints	126 (1.8%)
10 Other eye complaints		
11 Poor hearing/deafness	Ear complaints	134 (1.9%)
12 Tinnitus/noise in the ear		
13 Menieres discease/ear complaints causing balance problem		
14 Other ear complaints		
15 Stroke/cerebral haemorrhage/cerebral thrombosis	Heart & circulatory system	873 (12.4%)
16 Heart attack/angina		
17 Hypertension/high blood pressure/blood pressure		
18 Other heart problems		
19 Piles/haemorrhoids incl Varicose Veins in anus		
20 Varicose Veins/phlebitis in lower extremities		
21 Other blood vessels/embolic		
22 Bronchitis/emphysema	Respiratory system	603 (8.5%)
23 Asthma		
24 Hayfever		
25 Other respiratory complaints		
26 Stomach ulcer/ulcer (nes)/abdominal hemia/rupture	Digestive system	367 (5.2%)
27Other digestive complaints (e.g. stomach, liver, pancreas)		
28 Complaints of bowel/colon (e.ge large intestine, caecum, bowel)	Gentro-urinay system	139 (2.0%)
29 complaints of teeth/mouth, tongue		
30 kidney complaints		
31 Urinary tract infection		
32 Other bladder problems/incontinence		
33 Reproductive system disorders		
34 Arthritis/rheumatism/fibrositis	Musculoskeletal system	1,315 (18.6%)
35 Black problems/slipped disc .spine/neck		
36 Other Problems of bones/joints/muscles		
37 Infection and parasitic diseases	Infectious disease	16 (0.2%)
38 Disorders of blood and blood forming organs	Blood & related organs	46 (0.7%)
39 skin complaints	Skin complaints	102 (1.4%)
40 Other Complaints	other complaints	0 (0%)

For the purposes of the study, multimorbidity was defined as the presence of two or more longstanding conditions from different systems of the body.

#### Generating PW_HRQoL scores

In 2003 the SHeS included the SF-12 questionnaire, a self-reported measure of functional health status, which all adults aged 20 years and over were asked to complete. To generate a single PW_HRQoL score we used the SF-6D algorithm [9]. This algorithm involves preference-weighting 6 of the SF-12 question responses (3 physical health and 3 mental health) by the desirability for different health states. Preference-weights were derived from a survey representative of the UK general population (not the SHeS respondents) intended to enhance the generalisability of the results [9]. Summing across weighted question responses generates a single PW_HRQoL score for each respondent.

### Variables used in the analysis

We constructed a dataset containing respondents aged 20 years and over. Variables included: PW_HRQoL scores, respondent age, sex, presence of longstanding conditions, presence of multimorbidity, and socioeconomic deprivation as defined by the Scottish Index of Multiple Deprivation (SIMD) (http://www.scotland.gov.uk/Topics/Statistics/SIMD). SIMD is an aggregated measure of material deprivation derived from 37 indicators in seven domains (income, employment, health, education, access to services, housing and crime) and is determined at the data zone level (geographical areas with a median population of 769). Individuals in the SHeS 2003 are assigned a SIMD score based on their postcode of residence. SIMD scores ranged from 0 (least deprived) to 87.6 (most deprived). The Scottish Government defined cut-off values in the SIMD score to divide the general population into equally sized quintiles, used in its stratified sampling methodology. SIMD1 was defined as the least deprived quintile, with SIMD 2, 3, 4 and 5 denoting increasing levels of deprivation.

### Modelling approach

We created a dependent variable representing “ill health”, by subtracting PW_HRQoL scores from 1 (perfect health). Generalized Linear Modelling (GLM) was then used to test the suitability of different regression models. Following guidance by Glick et al. [17] we used the Modified-Park test to select the most appropriate model. Further, we applied the Akaike Information Criterion (AIC) and Bayesian Information Criterion (BIC) to assess model parsimony.

The most appropriate model specification was a Gaussian distribution with a link function, identified by the Modified-Park test. This model was also the most efficient, having the lowest AIC and BIC scores. This model is equivalent to ordinary least squares.

Three separate regression models were generated. First, we assessed the association between reductions in the PW_HRQoL score and increasing numbers of conditions (1, 2, 3+), measured as categorical variables. This was done for the sample as a whole and then stratified by quintiles of socioeconomic deprivation. The reference category was “no reported conditions”. The aim was to assess how PW_HRQoL declines as longstanding conditions accumulate. Following this analysis, we secondly assessed the association of multimorbidity, defined as 2+ conditions, with reductions in the PW_HRQoL score in the sample as a whole, and then stratified by quintiles of socioeconomic deprivation. Defining multimorbidity as 2+ conditions enabled statistical power to be largely maintained in the analysis. Finally, we assessed the associations between multimorbidity and reductions in HRQoL by age group (defined as 20–45, 45–64, 65+ years), for the sample as a whole and then stratified by quintiles of socioeconomic deprivation. For the second and third models, the reference category was “no multimorbidity” (i.e. 0 or 1 condition). All estimates were adjusted for sex and age; the latter modelled as a continuous variable.

## Results

### Scottish Health Survey (SHeS) 2003

The SHeS 2003 had a response rate of 77% and overall 11,442 individuals were surveyed. There was 23% missing households which were evenly spread across the general population, defined by SIMD. No bias was expected.

There were 7,754 individuals aged 20+ years asked to complete the SF-12. Of these, 7,054 individuals (91%) had a sufficient number of responses to generate Preference_Weighted Health Related Quality of Life (PW_HRQoL) scores and were included in the analysis (Table 2). Of the 9% of individuals excluded due to insufficient responses on the SF-12 the most deprived quintile was over represented. Excluded individuals were similar to those included in terms of age, sex and the presence of longstanding conditions. Given the large size of the dataset, we proceeded with a complete case analysis. No bias was expected, given the aim of the analysis was to assess how the impact of HRQoL from multimorbidity varied across deprived groups.

**Table 2:** Prevalence of longstanding conditions and multimorbidity in Scottish Health Survey 2003

	**No**. **of subjects**	**%**	**SIMD1**	**%**	**SIMD2**	**%**	**SIMD3**	**%**	**SIMD4**	**%**	**SIMD5**	**%**
Total sample	7,054	100%	1,362	19%	1,558	22%	1,609	23%	1,387	21%	1,139	16%
Age group												
20-44	2,819	40%	527	39%	601	39%	609	38%	610	44%	472	41%
(mean, sd)			(34.5, 6.6)		(34.9, 6.4)		(34.7, 6.8)		(33.1, 6.7)		(33.1, 7.3)	
45-64	2,639	37%	572	42%	591	38%	603	37%	463	33%	410	36%
(mean, sd)			(53.9, 5.4)		(54.1,5.6)		(54.5, 5.6)		(54.9,5.7)		(53.8,5.8)	
65+	1,596	23%	262	19%	366	23%	397	25%	314	23%	257	23%
(mean, sd, max)	(72.7, 6.2,95)		(72.7, 6.2,95)		(74.2, 6.4,96)		(73.7, 6.7,92)		(73.9, 6.5,95)		(73.7, 6.7,94)	
No of conditions												
0	3,937	56%	916	67%	895	57%	884	55%	717	52%	526	46%
1	1,875	27%	319	23%	417	27%	444	28%	386	28%	309	27%
2	822	12%	89	7%	161	10%	207	13%	177	13%	188	17%
3+	420	6%	38	3%	85	5%	74	5%	107	8%	116	10%
Rates of multimorbidity												
Total	1,242	18%	127	9%	246	16%	281	18%	284	21%	304	27%
20-44	216	8%	18	3%	37	6%	56	9%	46	8%	59	13%
45-64	505	19%	57	10%	93	16%	104	17%	119	26%	132	32%
65+	521	33%	52	20%	116	32%	121	31%	119	38%	113	44%

Table 2 shows the breakdown of the sample used in the analysis, including the prevalence of longstanding conditions and multimorbidity. 3,117 (45%) of survey respondents reported a history of one or more longstanding conditions, with 1,242 (18%) identified as multimorbid (2 or more conditions). The prevalence of multimorbidity was similar for men and women, and increased markedly with age. Overall, the prevalence was 3 times higher (27% vs 9%) in the most deprived quintile (SIMD5) compared to the least deprived quintile (SIMD1).

### Preference Weighted Health Related Quality of Life (PW_HRQoL) scores

**Figure 1:**
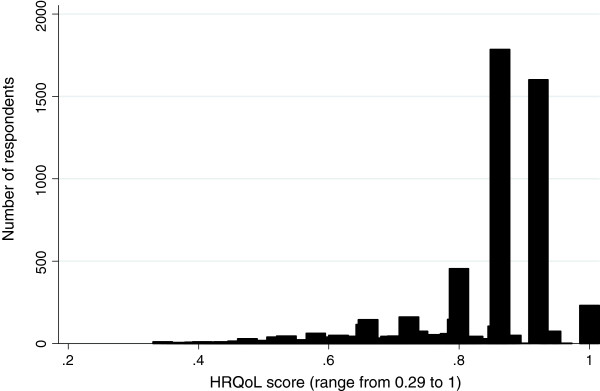
**Distribution of Health Related Quality of Life** (**HRQoL**) **scores in the Scottish Health Survey 2003.**

Figure 1 illustrates the distribution of PW_HRQoL scores with the survey population, with 1 indicating perfect health. The right skew demonstrates that the majority of the population reported relatively good health, with 64% of respondents reporting a PW_HRQoL score from 0.8 to 1, and the mean score was 0.84.

### Impact of increasing counts of longstanding conditions

**Figure 2:**
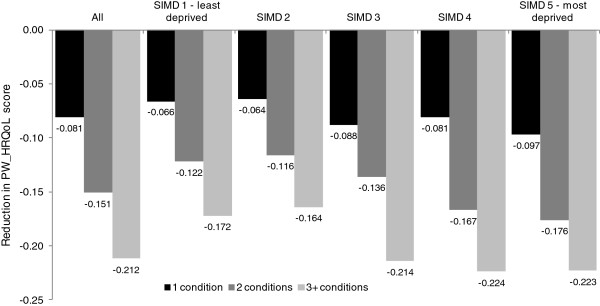
**Reduction in Health Related Quality of Life** (**HRQoL**) **score by number of conditions and quintiles of deprivation status**, **compared to people with no conditions.** *All p-values <0.0001.

Figure 2 illustrates that living with increasing numbers of conditions was associated with greater reductions in PW_HRQoL. In the general population, having a single condition was estimated to reduce the average PW_HRQoL score by 0.081, rising to 0.151 for 2 conditions (a fall of 0.07) and 0.212 for three of more conditions (a fall of 0.061). Therefore, there was a non-additive relationship: as conditions accumulative PW_HRQoL declines at a slower pace. Declines in PW_HRQoL were consistently greater in the most deprived quintile of the population. All estimates were statistically significant at the 1% level.

### Impact of multimorbidity

**Figure 3:**
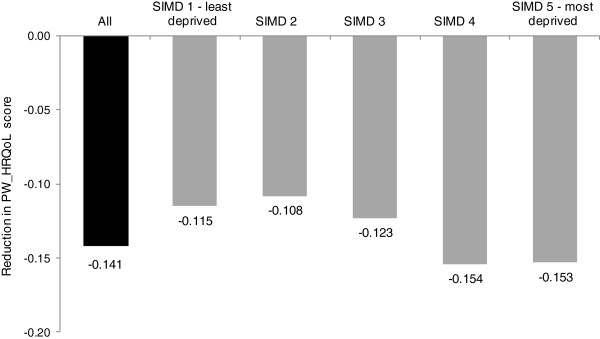
**Reduction in Health Related Quality of Life (HRQoL) score associated with multimorbidity by quintiles of deprivation status, compared to people without multimorbidity.** *All p-values <0.0001.

Figure 3 illustrates that living with multimorbidity (2 or more conditions) reduced the PW_HRQoL score by 0.141 in the general population. The impact of multimorbidity PW_HRQoL was 33% greater in the most deprived quintile (0.153) compared to the least deprived quintile (0.115) of the population. There was not a uniform gradient, with the fourth and fifth most deprived quintiles reporting similar falls in PW_HRQoL. All estimates were statistically significant at the 1% level.

### Impact of multimorbidity by age group

**Figure 4:**
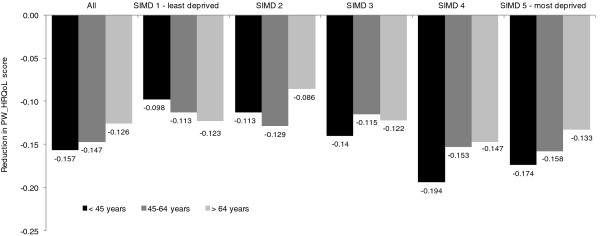
**Reduction in Health Related Quality of Life** (**HRQoL**) **score associated with multimorbidity by age group and quintiles of deprivation status**, **compared to people without multimorbidity.** *All p-values <0.0001.

Figure 4 shows that in the general population, the association of multimorbidity and lower quality of life was greatest in the youngest age group, with a reduction in PW_HRQoL score of 0.157 for the 20–44 year olds with multimorbidity, compared with 0.126 in the 65 and over age group with multimorbidity. For the least deprived quintile the age gradient was reversed. The most deprived quintile experienced more severe reductions in PW_HRQoL across all age groups compared to the least deprived quintile. This was most marked in the 20–44 year old age group where multimorbidity was associated with reductions of 0.178 compared to 0.098 respectively – a difference of 80%. This should be treated with caution, as within the SHeS just 18 people in the least deprived quintile were living with multimorbidity.

## Discussion

### Principal findings

This study has shown that multimorbidity is associated with substantial and progressive reductions in Health Related Quality of Life (HRQoL), as measured by a Preference_Weighted Health Related Quality of Life score (PW_HRQoL). This score provided a single summary measure of overall health, by weighting mental and physical states by their perceived importance as part of overall HRQoL. The use of a single score enables a simple and consistent assessment of the impact of conditions and how this varies across the population.

The clinical significance of multimorbidity is considerable. The PW_HRQoL measure used (SF-6D) ranges from 0.29 (worst health) to 1 (perfect health). The mean PW_HRQoL score was 0.84, and the average reduction in the score of respondents suffering from multimorbidity was 0.141 (compared to respondents with no or one longstanding condition) - a reduction of 17%.

Multimorbidity was associated with larger reductions in PW_HRQoL scores amongst participants living in the most deprived areas, with this deprivation effect most marked in younger multimorbid adults. While it is known that those living in the most deprived areas have a higher prevalence of multimorbidity of longstanding conditions [18], this is the first study to show that the impact of multimorbidity on PW_HRQoL score is also significantly greater in more deprived areas.

### Comparison with other studies

Previous studies also showed that PW_HRQoL falls markedly as the number of conditions increase. However, these studies were limited to six conditions (CVD and respiratory) [12,13]. Further, no analysis of the impact of multimorbidity conditional upon age and socioeconomic position was undertaken.

### Strengths and weaknesses

The SHeS 2003 included 40 longstanding conditions which is a greater amount than most previous studies [5,6]. The prevalence of obesity as an independent longstanding condition was not considered in the analysis. Obesity was not included in the SHeS definition of longstanding conditions, and the construction of a variable may have introduced double counting given several conditions included in the analysis are likely to be symptoms of obesity.

The inclusion of the SF-12 within the SHeS 2003 enabled a PW_HRQoL score to be estimated (using the SF-6D) permitting investigation into the association of multimorbidity and overall HRQoL, rather than separately on physical and mental health which most previous studies have done. Further, the size of the dataset and the comprehensive definition of socioeconomic deprivation (SIMD) meant that associations could be estimated by age group and quintiles of socioeconomic deprivation.

By using PW_HRQoL scores to estimate the burden of multimorbidity provides consistency with how economists measure the (HRQoL) burden of illness, termed health utilities; changes in which can be used in economic evaluation to assess the cost effectiveness of interventions. The clinical significance of multimorbidity as measured by PW_HRQoL demonstrates the potential need to develop interventions to lessen the impact of multimorbidity, rather than the traditional focus on single conditions.

The use of cross-sectional survey data limits the analysis to measurements of association. In addition, the SHeS does not provide data on duration or severity of conditions. These weaknesses are common to previous studies. A further weakness in national cross-sectional studies is that there is usually a poorer response rate within communities of lower socioeconomic position. We analysed the SHeS by quintiles of socioeconomic deprivation, as measured by the Scottish Index of Multiple Deprivation (SIMD). The most deprived quintile was underrepresented (16%) and this may have comprised representativeness, if for instance missing respondents were skewed toward the most deprived affecting case mix. However, the direction of any potential bias in our estimates is unknown.

A further potential limitation is that the sample was for 2003. It is possible that the nature of the relationship between PW_HRQoL, multimorbidity and deprivation may have changed over time. It is difficult to assess the likelihood of this limitation. The SHeS 2003 was the only SHeS survey that has included the SF-12 to enable this analysis to be undertaken.

### Future research

Further work could usefully focus on longitudinal studies to assess the causal impact of incurring longstanding conditions and developing multimorbidity throughout the life course [19,20]. Such studies could encompass both longitudinal cohort methods, and qualitative studies to better understand why the impact of multimorbidity varies by deprivation and age.

The hypothesis that the impact of multimorbidity on PW_HRQoL would be greater in more deprived groups compared to less deprived groups was largely corroborated. However, the deprivation gradient was not uniform, with the fourth and fifth most deprived quintiles having experienced similar declines in PW_HRQoL.

The general deprivation gradient may be the result of the existence of the ‘inverse care law’ [15], where the availability of services does not reflect the greater needs of deprived populations may result in insufficient health care resources to help people manage their conditions. Further, this may be compounded by deprived groups having reduced levels of personal capacity (e.g. reduced health literacy, agency) or community support available with which to manage or mitigate the impact of multimorbidity [14]. The impact of multimorbidity in younger (20–44 years) people in the most deprived quintile was 80% greater than those in the least deprived quintile. In addition, the deprivation gradient may (also) be due to case-mix where young deprived groups can suffer from greater mental health problems [21]. It is important to further investigate the associations between deprivation and multimorbidity to assess both its nature and the reasons underlying observed differences, in order to inform how best to address the deprivation gradient.

The inverse relationship between increasing age and the impact of multimorbidity may reflect greater levels of impairment due to multimorbidity in younger groups, or it may simply reflect that people of different ages adapt their expectations in different ways in response to living with chronic conditions. The reversal of this age gradient for the least deprived quintile needs to be explored further.

The paper stratified the SHeS into three age groups, with the eldest group 65 years and above. Due to statistical power concerns it was not possible to stratify age further. However, given that the incidence of multimorbidity increases further in older age groups [18], it is important, where possible, to investigate the consequent impacts on HRQoL.

The analysis focussed on the association between counts of conditions and HRQoL, rather investigating the impact of particular case-mix. A study by Fortin [5] found that different combinations of conditions are associated with different impacts on physical and mental health, as measured separately using the SF-36. Future work could usefully undertake a similar exercise exploring how case-mix impacts on an overall PW_HRQoL score (combining physical and mental health). Using a single score would permit systematic comparisons of the overall severity of case-mix, and identify patient groups who experience the greatest reductions in quality of life.

It is important to assess to what extent the reduction in PW_HRQoL from multimorbidity is actually modifiable to inform the development of interventions. Research could usefully investigate individual and community capacity to support effective self-management and adjustment to conditions.

Finally, estimating the burden of multimorbidity on PW_HRQoL would also provide consistency with how economic evaluation measures the impacts of interventions. PW_HRQoL scores (termed health utilities by economists) are used to generate quality adjusted life years (QALYs) by weighting length of life with quality of life, measured by the scores. Given that there has been limited research on this topic to date, assessing the burden of multimorbidity using PW_HRQoL scores in the general population may help strengthen the economic case for funding the development and implementation of interventions.

## Conclusion

Preference_Weighted Health Related Quality of Life (PW_HRQoL) decreases markedly with multimorbidity, and this is exacerbated by higher deprivation and younger age. There a need to prioritise interventions to improve quality of life for (especially younger) adults with multimorbidity in deprived areas.

## Competing interests

The authors declare that they have no competing interests.

## Authors’ contributions

KDL led on the design of the paper, undertook the analysis and wrote the first draft. EALF supervised the analysis. EALF, SW, EG, BG, and GCMW made substantial contributions to the interpretation and redrafting of the paper. All authors contributed to the intellectual content of the final draft and have approved the final draft.
